# The human immune response to saliva of *Phlebotomus alexandri*, the vector of visceral leishmaniasis in Iraq, and its relationship to sand fly exposure and infection

**DOI:** 10.1371/journal.pntd.0009378

**Published:** 2021-06-03

**Authors:** Ines Lakhal-Naouar, Rami Mukbel, Robert F. DeFraites, Rupal M. Mody, Lina N. Massoud, Dutchabong Shaw, Edgie M. Co, Jeffrey E. Sherwood, Shaden Kamhawi, Naomi E. Aronson

**Affiliations:** 1 Infectious Diseases Division, Uniformed Services University of the Health Sciences, Bethesda, Maryland, United States of America; 2 Department of Basic Veterinary Sciences, Jordan University of Science and Technology, Irbid, Jordan; 3 Department of Preventive Medicine and Biostatistics, Uniformed Services University of the Health Sciences, Bethesda, Maryland, United States of America; 4 Infectious Diseases Service, William Beaumont Army Medical Center, El Paso, Texas, United States of America; 5 Infectious Diseases Service, Walter Reed National Military Medical Center, Bethesda Maryland, United States of America; 6 Laboratory of Malaria and Vector Research, National Institute of Allergy and Infectious Diseases, Bethesda, Maryland, United States of America; Pasteur Institute of Iran, ISLAMIC REPUBLIC OF IRAN

## Abstract

**Background:**

Sand fly saliva exposure plays an important role in immunity against leishmaniasis where it has mostly been associated with protection. *Phlebotomus (Ph*.*) alexandri* transmits *Leishmania (L*.*) infantum*, the causative agent of visceral leishmaniasis (VL), in Iraq. Our group recently demonstrated that 20% of Operation Iraqi Freedom (OIF) deployers had asymptomatic VL (AVL) indicative of prior infection by the parasite *L*. *infantum*. Little is known about *Ph*. *alexandri* saliva, and the human immune response to it has never been investigated. Here, we characterize the humoral and cellular immune response to vector saliva in OIF deployers naturally exposed to bites of *Ph*. *alexandri* and characterize their immunological profiles in association to AVL.

**Methodology/Principal findings:**

The humoral response to *Ph*. *alexandri* salivary gland homogenate (SGH) showed that 64% of 200 OIF deployers developed an antibody response. To assess the cellular immune response to saliva, we selected a subcohort of subjects based on their post-travel (median 4 months; range 1–22 months) antibody response (SGH Antibody [Ab] positive or negative) as well as their AVL status; ten never-traveled controls were also included. Banked peripheral blood mononuclear cells (PBMC), collected ~10 years after end of deployment, were stimulated with SGH for 96 hours. The levels of IFN- γ, IL-6, IL-10, IL-13 and IL-17 were determined by ELISA. Our findings indicate that OIF deployers mounted a cellular response to SGH where the anti-SGH+ asymptomatic subjects developed the highest cytokine levels. Further, stimulation with SGH produced a mixture of pro-inflammatory and anti-inflammatory cytokines. Contrary to our hypothesis, we observed no correlation between the cellular immune response to *Ph*. *alexandri* SGH and prevention from asymptomatic infection with *L*. *infantum*.

**Conclusions/Significance:**

As we found, although all infected deployers demonstrated persistent disease control years after deployment, this did not correlate with anti-saliva systemic cellular response. More exposure to this vector may facilitate transmission of the *L*. *infantum* parasite. Since exposure to saliva of *Ph*. *alexandri* may alter the human immune response to bites of this vector, this parameter should be taken into consideration when considering the VL risk.

## Author summary

This is the first report of human immune responses to *Phlebotomus alexandri*. *Phlebotomus alexandri* is a sand fly found in Southwest Asia and is the vector for transmission of the parasite *Leishmania infantum*, agent of visceral leishmaniasis. During the bite of this sand fly, a small amount of saliva is injected into the skin. In this study, we report the human immune response to *Phlebotomus alexandri* saliva. We found that 64% of people who traveled to endemic Iraq developed antibodies directed toward this sand fly’s saliva. This suggests that saliva is very immunogenic and that anti-saliva immune responses could be a good indicator of vector exposure. Additionally, we studied the cellular immune responses in saliva-stimulated white blood cells and found a Th2 biased cytokine profile.

## Introduction

Visceral leishmaniasis (VL), due to *Leishmania infantum*, is a persistent intracellular parasitic infection transmitted by the bite of infected sand flies. VL is endemic in central Iraq and has expanded to southeastern regions after the Gulf War [1]. In Iraq, the *Leishmania* (*L*). *infantum* parasite is transmitted by the bite of an infected *Phlebotomus (Ph)*. *alexandri* sand fly [2,3]. In 2003–4, a 0.09% *L*. *infantum* infection rate was reported for *Ph*. *alexandri* at Tallil Air Base, Iraq [4]. Asymptomatic visceral leishmaniasis (AVL), indicative of infection with *L*. *infantum*, showed a substantial prevalence in Operation Iraqi Freedom (OIF) deployers. Using molecular and immunological assays on blood samples, we showed that 20% of OIF deployers (n = 200) had asymptomatic *L*. *infantum* infection defined by a positive cellular/humoral response or nucleic acid testing result [5]. Although *Ph*. *alexandri* is anthropophilic, it is not peridomiciliary and is mostly found in outdoor environments [6,7]. In mountainous areas, the density of sand flies is decreased and restricted to places with sporadic cottages or stables [8]. A shift in peak activity from early evening in April and October to later in the night during the hotter months of May and June was observed for several sand fly species in Iraq, including *Ph*. *alexandri*, likely increasing the rate of bites during sleep [9].

Compared to several sand fly species such as *Lutzomyia (Lu*.*)*. *longipalpis*, the New World VL vector [10], and *Ph*. *papatasi* and *Ph*. *perniciosus*, Old World vectors of cutaneous leishmaniasis (CL) and VL respectively [11,12], little is known about *Ph*. *alexandri* and the immunogenicity of its saliva in humans. Exposure to *Phlebotomus* species bites or salivary proteins results in strong cellular and/or humoral immunity specific to some of these components [13,14]. Experimental exposure of naïve hosts (including mice [15], dogs [16] and humans [17]) to sand fly bites shows that antibody responses to saliva are acquired rapidly, usually within a few weeks of exposure and that the magnitude of the antibody response increases with the number of sand fly bites [16]. Field studies have also demonstrated the presence of anti-saliva antibodies in humans naturally exposed to sand fly bites [15,18]. Antibodies against sand fly saliva or against salivary recombinant proteins may serve as markers of vector exposure [19,20] as well as potential surrogate markers for the risk of *Leishmania* infection [21].

Regarding human cellular immune responses to sand fly saliva, human volunteers experimentally exposed to *Lu*. *longipalpis* bites displayed an increased frequency of CD4^+^ and CD8^+^ T cells as well as an increase in IFN-γ and IL-10 production upon *in vitro* PBMC stimulation with vector salivary gland homogenate (SGH) [17]. Analysis of cellular immune responses against *Ph*. *papatasi* saliva in PBMC from individuals naturally exposed to bites of this vector showed low proliferation, absence of IFN-γ production but significant IL-10 levels, which could favor establishment of infection with *L*. *major* [22]. Mali individuals exposed to bites of the CL vector *Ph*. *duboscqi* displayed a systemic immune response to its saliva involving the production of Th1 and Th2 cytokines; in contrast an abundant local expression of IFN-γ in the absence of a Th2 response was observed in skin biopsies of bite sites from individuals with a positive delayed-type hypersensitivity reaction [23].

Cellular and humoral responses to *Ph*. *alexandri* saliva have not been reported to date. This vector species has not been raised in insectaries, necessitating use of wild-caught sand flies for our work. Here, we report an analysis of cellular and humoral responses directed against *Ph*. *alexandri* saliva in Iraq-deployed U.S. personnel, presumed immunologically-naïve to vector saliva prior to deployment. We also investigate saliva-specific responses in individuals with or without AVL; notably in this cohort there were no participants with active VL [5].

## Materials and methods

### Ethics statement

This research protocol was approved by the Uniformed Services University (USU), Walter Reed National Military Medical Center (WRNMMC), and the William Beaumont Army Medical Center (WBAMC) Institutional Review Boards (IRB). All participants provided written informed consent.

### Characterization of sample and data collection sources

The AVL study cohort, further described in [5], included 200 OIF deployed asymptomatic individuals and 46 never-traveled healthy control subjects (HC, living in US, and have never been to a leishmaniasis endemic area lifelong). To study the long-lasting cellular responses, peripheral blood mononuclear (PBMC) cells were isolated from blood collected on average a decade after deployment; however the humoral response to SGH was assessed using banked sera from the same 200 OIF deployers, collected a median of 4 months (range 1–22 months) after return from deployment [5]. These sera were obtained from the Department of Defense Serum Repository, The Armed Forces Health Surveillance Branch, U.S. Department of Defense, Silver Spring, MD; release dates 2015–2017. To study the cellular immune responses to *Ph*. *alexandri* SGH, we selected a subcohort of 82 individuals (41% of deployers) and 10 never traveled controls based on residual cell availability (banked cells) to perform all required testing, reactivity in SGH ELISA, and AVL status. Using PBMC, AVL status was predetermined [5] as having either an elevated interferon gamma level post-stimulation with soluble *Leishmania* antigens (SLA), a positive SLA antibody response, or a positive *L*. *infantum* PCR (REPL target) [24]. For this study, we classified the selected subcohort into four groups (Table 1): AVL^-^SGHAb^+^ (Ab: Antibody) (n = 35) (AVL^-^: deployers who did not have evidence of exposure to *Leishmania*), AVL^+^SGHAb^+^(n = 28) (SGHAb^+^: presence of antibody response to SGH), AVL^+^SGHAb^-^ (n = 9), AVL^-^SGHAb^-^(n = 10) and included never traveled controls (n = 10). Sand fly exposure information was abstracted from the AVL leishmaniasis risk factor survey [5].

**Table 1:** Demographic Characteristics of the Study Population.

		SGHAb+		SGHAb-		Controls
		AVL-	AVL+	AVL-	AVL+	
**Sample size (n)**		35	28	10	9	10
**Gender**						
	Male	29	26	8	7	7
	Female	6	2	2	2	3
**Race**						
	Caucasian	26	23	9	7	8
	African- American	7	4		1	
	Asian	2		1		2
	PI		1			
	Other				1	
**Age**						
	Mean (yrs.)	42.8	41	40.7	39.4	28.5*
**Service Branch**						
	Army	31	26	9	7	
	Navy	1	0	0	2	
	Marines	3	2	1	0	

SGH: Salivary Gland Homogenates; Ab: Antibody, AVL: asymptomatic visceral leishmaniasis; yrs.: years; PI: Pacific Islander, +: positive; -: negative

*p<0.0001, ANOVA

### *Ph*. *alexandri* collection and SGH preparation

*Ph*. *alexandri* sand flies were collected using CDC light traps (John W. Hock Company) during the summers of 2016 and 2017 at a site on the outskirts of Waqqas, Jordan located in the northern Jordan Valley, GPS coordinates 32.547852 N, 35.614674 E. Waqqas is a small city in the Irbid governorate with an average annual rainfall of 250 ml. *Ph*. *alexandri* females were identified using pharyngeal and spermathecal characteristics [25]. Salivary glands from female *Ph*. *alexandri* were dissected and transferred to polypropylene vials, usually in groups of 20 pairs of glands in 20 μl of HEPES-saline. Salivary glands were stored at −80°C until needed, when they were disrupted by sonication and prepared as previously described [5].

### Determination of anti-SGH antibody response

Banked sera were analyzed using an indirect enzyme-linked immunosorbent assay (ELISA) that measures anti*-Ph*. *alexandri* SGH total IgG as previously reported [5]. The ELISA cutoff was defined as average OD of unexposed controls + 3SD (0.108).

### Cell stimulation and cytokine quantification

Banked PBMC were thawed and rested overnight at 37°C, 5% CO_2_. One million cells were plated in triplicate and stimulated with 1ug/ml *Ph*. *alexandri* SGH, 10% phytohemagglutinin (PHA, Life Technologies) or left unstimulated. Ninety-six hours later, supernatants were collected and stored at -80°C for cytokine quantification. Levels of IFN-γ, IL-4, IL-6, IL-10, IL-12p70, IL-13 and IL-17 in the supernatants were determined by ELISA using commercial cytokine kits (Uncoated human ELISA kits, Invitrogen) as specified by the manufacturer.

### Statistical analysis

Cytokine levels were compared using a nonparametric Wilcoxon test and a Pearson correlation analysis. All tests were performed using Prism software (GraphPad Software, Inc.). Differences were considered significant if p value < 0.05. For the analysis of risk factors for sand fly exposure, differences in categorical variables were analyzed using Fisher’s exact or Chi squared tests; continuous variables were assessed using Student’s t-test or analysis of variance (ANOVA). Odds ratios are stated as point estimates with 95% Confidence Intervals (95% C.I.)

## Results

### Individuals deployed to *L*. *infantum* endemic Iraq developed antibodies against *Ph*. *alexandri* saliva

**Fig 1:**
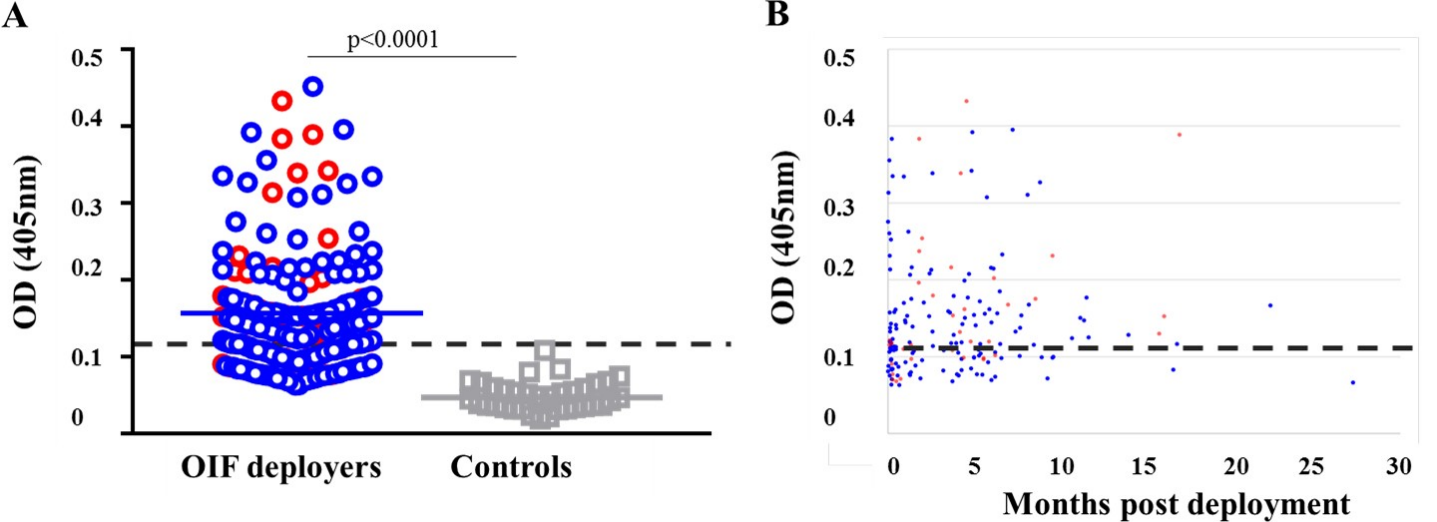
The humoral response to SGH of *Phlebotomus alexandri* (A) ELISA reactivity of banked sera from OIF deployers (n = 200) and never traveled controls (n = 46) against SGH. (B) Anti-SGH total IgG levels of OIF deployers measured at a median of 4 (range 1–22) months post-deployment (once per subject). Dotted black line shows the cutoff value, which is defined as the mean OD + 3 SDs for the values obtained with sera from never-traveled controls (0.108). AVL subjects are represented in red dots, blue dots are subjects who deployed but were not infected (AVL^-^) and grey squares are never traveled controls. The significance of differences between groups was evaluated by the Mann-Whitney test. P values of <0.05 were considered significant. Solid lines indicate mean OD values.

We examined the antibody levels to *Ph*. *alexandri* SGH in serum samples of 200 Iraq deployed individuals and 46 control individuals [5]. Sixty four percent of OIF deployers had an antibody response to *Ph*. *alexandri* saliva (Fig 1A) (accounting for 77% AVL^+^ and 61% AVL^-^) (p = 0.06, Odds Ratio 2.14 [95% CI 0.95, 4.81]). We also evaluated the persistence of this humoral response by measuring antibody reactivity to SGH after OIF deployers had returned to the United States where *Ph*. *alexandri* is absent. Interestingly, antibodies to sand fly saliva could be detected in a few subjects as late as 18 months post exposure to bites of *Ph*. *alexandri* (Fig 1B).

### The systemic immune response to *Ph*. *alexandri* SGH is skewed toward a Th2 profile

To assess the cellular immune response to *Ph*. *alexandri* saliva, we selected a subcohort of 82 OIF deployers and 10 controls (Table 1). The selected subcohort consisted mainly of male individuals (81.7%) of white race or ethnicity (79.2%) and a median age of 40.9 (range 24–61) years. Most served in the Army (89%). The average number of days spent in Iraq was 300 (range 30–790). Controls were mainly white (70%) males with a median age of 28.5 years (range 23–35) (Table 1). These demographics were closely aligned with the overall cohort characteristics except the age of the control group is significantly different than each of the deployed groups (ANOVA p < .0001, Tukey’s HSD p < .01) [5]. The OIF deployers were sorted into AVL^-^SGHAb^+^, AVL^+^SGHAb^+^, AVL^+^SGHAb^-^ and AVL^-^SGHAb^-^ groups based on their antibody reactivity to vector saliva and AVL status.

**Fig 2:**
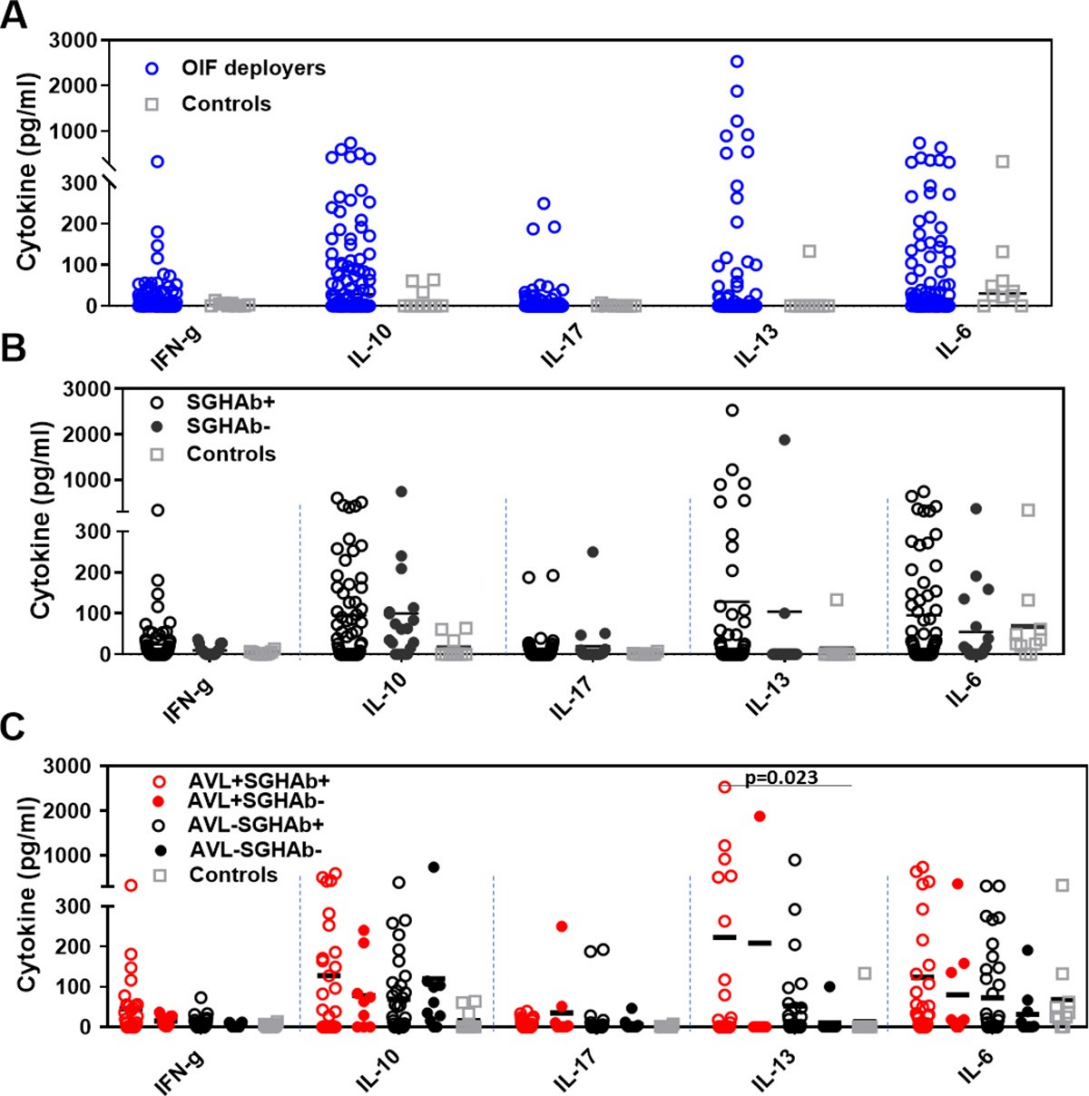
Cytokine levels induced by *Ph*. *alexandri* salivary gland homogenate after 96 hours stimulation. Unless specifically shown, none of the comparisons reached statistical significance. (A) Response in OIF deployers (open blue circles) versus never traveled controls (open grey squares). (B) Response in SGHAb^+^ (black open circles), SGHAb^-^ deployers (solid black circles) and never traveled controls (open grey squares). (C) Response in AVL^+^SGHAb^+^ (open red circles), AVL^+^SGHAb^-^ (solid red circles), AVL-SGHAb^+^ (open black circles), AVL-SGHAb^-^versus never traveled controls (open grey squares). Cytokines were quantified by ELISA in the culture supernatants of PBMCs stimulated with *Ph*. *alexandri* salivary gland extract (1 ug/ml) during 96h. The significance of differences between groups was evaluated by the Mann-Whitney test. P values of <0.05 were considered significant. Horizontal lines represent mean values.

Stimulation of banked PBMCs from 82 OIF deployers with *Ph*. *alexandri* SGH produced variable and higher amounts of IFN-γ, IL-6, IL-13, IL-10 and IL-17 compared to control subjects (Fig 2A). Though not statistically significant, potentially due to the variability of the response in OIF deployers, this indicates prior contact with *Ph*. *alexandri*. No IL-4 or IL-12p70 were detected. Next, we compared the cellular response in OIF deployers with or without a anti-SGH antibodies(likely indicative of a significant or weak/absent exposure to bites by *Ph*. *alexandri*, respectively). The SGHAb^+^ group consisted of 35 individuals selected for the highest serum total IgG values against *Ph*. *alexandri* SGH (group average OD = 0.23). Levels of most SGH-stimulated cytokines were higher in those who were seropositive to *Ph*. *alexandri* saliva compared to those who were seronegative (SGHAb^-^), with the exception of IL-10 and IL-17 (Fig 2B). The fold increase in the mean cytokine levels over controls were 2.62, 1.23 and 1.75-fold higher for IFN-γ, IL-13 and IL-6, respectively, in SGHAb^+^ compared to SGHAb^-^ group. The mean levels of IL-10 were similar in both groups while IL-17 levels were 2-fold lower in the SGHAb^+^ group (Fig 2B).

After defining the profile based on reactivity to SGHAb, we further compared the cytokine response in terms of AVL status (AVL^+^ or AVL^-^ individuals) (Fig 2C). Among the studied groups, the AVL^+^SGHAb^-^ had a relatively poor response, while the magnitude of the cellular immune response against saliva was highest in the AVL^+^SGHAb^+^ group and consisted mainly of IL-6, IL-10 and IL-13. AVL^+^SGHAb^+^ produced a significantly higher level of IL-13 (p = 0.023) compared to HC (Fig 2C). Though, a statistically significant difference was observed for IL-13 for AVL^+^SGHAb^-^ versus AVL^-^SGHAb^+^ (p = 0.0039) and AVL^+^SGHAb^-^ versus HC (p = 0.02), this was caused by an outlier responder and is not considered representative of the group response (Fig 2C). Moreover, we saw a 4-fold increase in IFN-γ for AVL^+^SGHAb^+^ (mean 40 pg/ml) compared to AVL^-^SGHAb^+^ group (mean 10 pg/ml), though the difference was not statistically significant.

Based on the proinflammatory (IFN-γ and IL-17) or anti-inflammatory (IL-10 and IL-13) nature of the secreted cytokines, the AVL^+^SGHAb^+^ individuals were more likely to show a mixed response while the AVL^-^SGHAb+ group was more polarized towards either a proinflammatory or anti-inflammatory response (Table 2). Analyzing the ratio of IFN-γ to IL-10 or IL-13 indicates that the response is overall biased towards an anti-inflammatory profile (S1 Table). Independent of the four analyzed subgroups, 62% (51/82) of deployed individuals developed a cellular response to SGH, of which 50% (26/51) were AVL^+^ (Table 2). Individual responses as shown for Table 2 are masked by the group mean.

**Table 2:** Nature of the Cellular Immune Response Based on SGH Antibody Response and Asymptomatic Visceral Leishmaniasis Status.

	SGHAb+		SGHAb-	
	AVL-	AVL+	AVL-	AVL+
**Number**	35	28	10	9
Pro-inflammatory*	9 (25.7%)	6 (21.4%)	2 (20%)	3 (33.3%)
Anti-inflammatory**	9 (25.7%)	4 (14.2%)	4 (40%)	1 (11.1%)
**Mixed**	1 (2%)	11 (39.2%)	0 (0%)	1 (11.1%)
**None**	16 (45.7%)	7 (25%)	4 (40%)	4 (44.4%)

*Pro-inflammatory: IFN-γ and IL-17

**anti-inflammatory: IL-10 and IL-13

Having defined the cytokine profile after stimulation with *Ph*. *alexandri* SGH, we speculated whether it may be affected by, or may affect, the response to *Leishmania*. To address this, we analyzed the overall response to the SGH in the context of infection with *L*. *infantum* based on the historical cellular response of PBMC stimulated with soluble *Leishmania infantum* antigen (SLA) as demonstrated in a previous study [5]. Twenty of the 26 (76.9%) AVL^+^ individuals with a cellular response to SGH and 6/11(54.5%) AVL^+^ but no cellular SGH response were also *Leishmania infantum* IFN-γ release assay-positive (IGRA^+^).

### Risk factors for sand fly exposure

Risk characteristics for sand fly exposure were assessed in the AVL-SGHAb^+^, AVL^+^SGHAb^-^, AVL^+^SGHAb^+^ and AVL^-^SGHAb^-^ groups (Table 3). A statistical difference in terms of increased mean *Ph*. *alexandri* total IgG antibody levels was found between these groups, (p < .0001) (Table 3). No statistical association was found comparing the use, the frequency of use, of an insect repellent, or permethrin–treated uniform usage with either AVL or SGHAb status (Table 3). Though not statistically significant, the AVL^-^SGHAb^-^ group appears to have slept fewer nights in open environment without shelter (9.2 nights) compared to all other groups (34.9–54.1 nights), suggesting that spending less time outdoors had the most protective effect against sand fly bites and therefore the risk of contracting VL.

**Table 3:** Associations of Reported Sand Fly Exposure Risk Factors to SGH Antibody Levels and Diagnosis of Asymptomatic Visceral Leishmaniasis.

	SGHAb+		SGHAb-	
	AVL-	AVL+	AVL-	AVL+
**Number**	**35**	**28**	**10**	**9**
Sand fly saliva antibody ELISA O.D. (avg.)*	**0.2388**	**0.2026**	**0.0733**	**0.0934**
**Average # days in Iraq**	**288**	**322**	**290**	**290**
**Avg. nights in open, no shelter**	**76.3**	**98.9**	**13.1**	**81.2**
**Avg. nights in shelter without a/c**	**99.9**	**80**	**155**	**114.7**
**Avg. nights with a/c**	**126.5**	**98.8**	**132.9**	**169.4**
**Typical summer sleepwear n(%)**				
**Full uniform +/_ Boots**	**5 (15)**	**3 (11)**	**1 (10)**	**4 (44)**
**Shorts and T-shirt (Physical fitness uniform)**	**19**	**18**	**5**	**4**
**“Skivvies” only**	**7 (21)**	**5 (18)**	**3 (30)**	**1 (11)**
**Other**	**3**	**2**	**1**	**0**
**Exposure to dogs**				
**Had a local pet**	**4**	**2**	**2**	**2**
**Others in unit had a pet**	**13**	**10**	**5**	**4**
**Worked with MWDs**	**4**	**2**	**1**	**1**
**Little/no direct contact n(%)**	**16 (46)**	**17 (61)**	**5 (50)**	**5 (56)**
**^more than one type response allowed**				
**Insect repellent**				
**Used None n (%)**	**13 (37)**	**10 (36)**	**5 (50)**	**3 (33)**
**Brought own brand**	**2**	**3**	**0**	**1**
**Used military issued repellent**	**13**	**7**	**1**	**4**
**Used both**	**7**	**8**	**4**	**1**
**Frequency of repellent use**				
**Never n (%)**	**12 (34)**	**11 (39)**	**5 (50)**	**3 (33)**
**≤ 1/week**	**17**	**9**	**2**	**4**
**>1/week**	**4**	**4**	**3**	**1**
**Daily n (%)**	**2 (6)**	**4 (14)**	**0**	**1 (11)**
**Permethrin–treated uniforms**				
**None n (%)**	**12 (34)**	**10 (36)**	**1 (10)**	**4 (44)**
**Unknown**	**3**	**1**	**1**	**0**
**Some uniforms treated**	**9**	**10**	**3**	**2**
**All uniforms treated n(%)**	**11 (31)**	**7 (25)**	**5 (50)**	**3 (33)**

*Annova p < .0001

SGH: Salivary Gland Homogenates; AVL: Asymptomatic Visceral Leishmaniasis, Ab: Antibody, OD: optical density, a/c: air conditioning

## Discussion

The sand fly bite is a critical event in *Leishmania* transmission and vector saliva is a determining factor for *Leishmania* infection. The large array of pharmacological substances in sand fly saliva is essential for efficient blood feeding by the sand fly, promoting blood flow to the bite site and modulating the local immune response to the injected molecules, thus interfering with both the host hemostatic and immune responses [14,26]. Sand fly saliva has immunomodulatory properties due to antigenic components, mainly proteins [10]. The composition of sand fly saliva differs between vector species, but sometimes differences can also be detected among similar species from distinct geographical areas [27]. In this study, we analyzed the humoral and cellular immune responses directed against the saliva of wild-caught *Ph*. *alexandri*, a vector of VL, in persons deployed to *L*. *infantum* endemic regions in Iraq. We investigated how natural exposure to *Ph*. *alexandri* shapes the human immune response to salivary gland antigens and whether such immune responses are associated with AVL. Despite the study limitation of not knowing when AVL+ individuals were exposed to a *L*. *infantum*-infected bite amid recurrent uninfected bites, we achieved our objective by analyzing the cellular response to wild caught *Ph*. *alexandri* saliva in well-defined groups (AVL^-^SGHAb^+^, AVL^+^SGHAb^+^, AVL^+^SGHAb^-^, AVL^-^SGHAb^-^, and HC) and presented our findings in the context of self-reported risk factors for sand fly exposure.

A seropositive response to *Ph*. *alexandri* SGH was detected in 64% of 200 Iraq-deployed individuals, mirroring earlier findings regarding the immunogenicity of saliva from other sand fly vectors in endemic populations, including *Lu*. *intermedia* (59%, mainly IgG1 and IgG4) [28], *Ph*. *papatasi* (40%) [29] and *Lu*. *longipalpis* [10]. The antibody response elicited by sand fly salivary proteins has been shown to be specific for several vector species [27] but can be found in common for closely related species that share high levels of similarity in their salivary proteins like *Ph*. *duboscqi* and *Ph*. *papatasi* [30]. Additionally, a high titer of anti-saliva antibodies has been associated with an increased risk of contracting leishmaniasis due to more frequent contact with sand flies, thus increasing the probability to encounter infected sand fly bites [15,19].

In endemic areas, the sand fly population fluctuates seasonally which may also influence host anti-saliva antibody or cellular levels [6,31]. Importantly, after a six-month or one-year bite-free period, boosting by re-exposure with *Lu*. *longipalpis* or *Ph*. *argentipes* bites, respectively, caused significant increases of antibody levels in humans indicating an antibody memory response to SGH for both sand fly species [27]. In our study, antibodies to SGH could be detected up to 18 months after last exposure in at least five individuals (among 128) including three with AVL. Those subjects had more than a one-time deployment suggesting that re-exposure may be associated with longer persistence in antibody levels.

In terms of cellular responses to *Ph*. *alexandri* saliva, we demonstrated that a high amount of IL-10 was produced following SGH stimulation in the deployed groups, regardless of their antibody response to SGH. We hypothesize that some study subjects may have received a low number of sand fly bites that are insufficient to induce a robust adaptive immune response. In naïve individuals lacking an adaptive immune response to sand fly saliva, immunomodulatory salivary proteins have been shown to be mostly anti-inflammatory [31]. Although no data on the composition of *Ph*. *alexandri* saliva, and no ‘omics’ studies exist yet, we speculate that IL-10 secretion possibly results from the presence of adenosine and adenosine monophosphate in sand fly saliva [32,33]. Adenosine increases IL-10 and decreases IL-12 production [34]. Alternatively, individuals with a negative history to any type of *Leishmania* infection but that were exposed to *Ph*. *papatasi* or *Lu*. *intermedia* also developed an IL-10 dominant response [22,28], which inhibits the proliferation of lymphocytes producing IFN-γ. Following *Ph*. *papatasi* bites, CD8^+^ T cells were the primary source of IL-10 [22]. In contrast, the source of IL-10 after *Lu*. *intermedia* exposure was both CD4^+^CD25^+^Foxp3^+^ and Foxp3^−^ cells [28]. IL-10 has suppressive roles that could help limit the immune-mediated VL pathology, especially in the liver, and promote parasite replication and disease progression [35].

In addition to IL-10, both IL-6 and IL-13 were produced in response to *Ph*. *alexandri* SGH stimulation of PBMC from our volunteers who deployed to a VL endemic area nearly a decade ago. Interleukin-6 is a multifunctional cytokine produced by several cell types and has a systemic effect on the immune system (primarily on B lymphocytes). It also stimulates hematopoiesis [36]. We speculate that saliva of *Ph*. *alexandri* has an inhibitory effect on lymphocyte proliferation suppressing early production of IL-2, IL4, and IFN-γ and increasing production of IL-6, similar to what was reported for *Lu*. *longipalpis* and *Ph*. *papatasi* saliva [30,37]. *Lu*. *longipalpis* saliva induced an increase in IL-6, IL-8, and IL-12p40 production by PMBCs but decreased TNF-α and IL-10 production [38]. However, increased levels of IL-10 mRNA were observed in the ear tissues of mice co-injected with parasites and SGH of *Lu*. *longipalpis* that was associated with higher levels of IL-10 in supernatants of restimulated draining lymph node (LN) cells [39].

In terms of the cellular response profile to *Ph*. *alexandri* saliva in the context of AVL status, the AVL^+^SGHAb^+^ showed the highest response, producing a mix of proinflammatory and anti-inflammatory responses. In comparison, AVL^-^SGHAb^+^ individuals were more polarized towards either a proinflammatory or anti-inflammatory response that may influence their infection outcome upon future exposure to a *Leishmania*-infected bite. The mixed or dichotomous response of human PBMC upon stimulation with vector SGH was also observed in individuals chronically exposed to *Ph*. *duboscqi* saliva who were categorized as Th1, Th2, or mixed responders [23]. Additionally, individuals experimentally exposed to *Lu*. *longipalpis* bites produced systemic IFN-γ, IL-10, and TNF-α [17]. The majority of the SGHAb^+^ subgroup was also *Leishmania* IGRA^+^ [5] which raises the possibility that immunity to saliva may produce an environment that helps the parasites survive or persist. Alternatively, another explanation could be that multiple bites over time are needed to develop SGH antibody responses (based on controlled human challenge studies) and that the SGHAb^+^ group was selected for a higher degree of vector saliva exposure and potentially greater likelihood for encountering a *Leishmania* infected bite.

Collectively, our findings highlight differences in the bite-induced immune responses to saliva of wild *Ph*. *alexandri* in this American military cohort. We observed mixed Th1 and Th2-biased responses to saliva that may result in different individual outcomes following exposure to an infected bite. This observation is further complicated by the fact that we do not know the discrete timing/order of the uninfected sand fly exposure versus *Leishmania* inoculation in our subjects and also that we analyzed the systemic and not the cellular immune response at the skin level. A local immune analysis at the skin bite site may provide additional clues and could be more appropriate for associating saliva-specific immunity to protection after sand fly exposure. Moreover, expecting that Th1-biased or mixed immune responses to vector saliva can prevent infection is a high bar, it might have contributed to an appropriate immunologic response which prevented active VL. Future studies can better address the effect of varied immune responses to vector saliva on the outcome of VL in a larger cohort that includes symptomatic VL cases. Finally, our convenience samples came from banked sera collected close in time to end of deployment (since humoral responses to SGH wane) while PBMC were collected on average about a decade later. We assume that persistent cellular memory immune responses against salivary homogenate will be the most impactful in preventing VL activation.

It was shown that mounting a humoral immune response to *Lu*. *longipalpis* saliva paralleled the development of a cell-mediated immune response to *Leishmania* [6]. In our study, no such correlation was observed. The absence of correlation between proliferative and humoral response to *Ph*. *papatasi* saliva has also been reported [11]. On the other hand, our results suggest that more intense or longer exposure to bites amplify the chances of getting an infected bite thus favoring AVL development.

Taken together, our study highlights the complex nature of the human immune response to sand fly saliva and its influence on the resulting immunity agains*t Leishmania*. Future studies should further explore the relevant antigens in *Ph*. *alexandri* saliva and their contribution to immunogenicity and susceptibility to, or protection from, *Leishmania* infection.

## Supporting information

S1 Table Ratio IFN-γ to IL-10, IL-13 and IL-17. SGH: Salivary Gland Homogenates; AVL: asymptomatic visceral leishmaniasis
(DOCX)

## References

[1] Salam N, Al-Shaqha WM, Azzi A. Leishmaniasis in the middle East: incidence and epidemiology. PLoS Negl Trop Dis. 2014;8(10):e3208. Epub 2014/10/03. doi: 10.1371/journal.pntd.0003208 ; PubMed Central PMCID: PMC4183486. 25275483 PMC4183486

[2] Azizi K, Rassi Y, Javadian E, Motazedian MH, Rafizadeh S, Yaghoobi Ershadi MR, et al. Phlebotomus (Paraphlebotomus) alexandri: a probable vector of Leishmania infantum in Iran. Ann Trop Med Parasitol. 2006;100(1):63–8. Epub 2006/01/19. doi: 10.1179/136485906X78454 . 16417715

[3] Stoops CA, Heintshcel B, El-Hossary S, Kaldas RM, Obenauer PJ, Farooq M, et al. Sand fly surveillance and control on Camp Ramadi, Iraq, as part of a leishmaniasis control program. J Vector Ecol. 2013;38(2):411–4. Epub 2014/03/04. doi: 10.1111/j.1948-7134.2013.12059.x . 24581374

[4] Coleman RE, Hochberg LP, Swanson KI, Lee JS, McAvin JC, Moulton JK, et al. Impact of phlebotomine sand flies on U.S. military operations at Tallil Air Base, Iraq: 4. Detection and identification of leishmania parasites in sand flies. J Med Entomol. 2009;46(3):649–63. Epub 2009/06/06. doi: 10.1603/033.046.0333 . 19496439

[5] Mody RM, Lakhal-Naouar I, Sherwood JE, Koles NL, Shaw D, Bigley DP, et al. Asymptomatic Visceral Leishmania infantum Infection in US Soldiers Deployed to Iraq. Clin Infect Dis. 2019;68(12):2036–44. Epub 2018/09/22. doi: 10.1093/cid/ciy811 ; PubMed Central PMCID: PMC6769235. 30239631 PMC6769235

[6] Maroli M, Feliciangeli MD, Bichaud L, Charrel RN, Gradoni L. Phlebotomine sandflies and the spreading of leishmaniases and other diseases of public health concern. Med Vet Entomol. 2013;27(2):123–47. Epub 2012/08/29. doi: 10.1111/j.1365-2915.2012.01034.x . 22924419

[7] Colacicco-Mayhugh MG, Masuoka PM, Grieco JP. Ecological niche model of Phlebotomus alexandri and P. papatasi (Diptera: Psychodidae) in the Middle East. Int J Health Geogr. 2010;9:2. Epub 2010/01/22. doi: 10.1186/1476-072X-9-2 ; PubMed Central PMCID: PMC2823717. 20089198 PMC2823717

[8] Ebrahimi S, Bordbar A, Rastaghi AR, Parvizi P. Spatial distribution of sand fly species (Psychodidae: Phlebtominae), ecological niche, and climatic regionalization in zoonotic foci of cutaneous leishmaniasis, southwest of Iran. J Vector Ecol. 2016;41(1):103–9. Epub 2016/05/28. doi: 10.1111/jvec.12200 . 27232131

[9] Coleman RE, Burkett DA, Sherwood V, Caci J, Spradling S, Jennings BT, et al. Impact of phlebotomine sand flies on U.S. Military operations at Tallil Air Base, Iraq: 2. Temporal and geographic distribution of sand flies. J Med Entomol. 2007;44(1):29–41. Epub 2007/02/14. doi: 10.1603/0022-2585(2007)44[29:iopsfo]2.0.co;2 . 17294918

[10] Gomes R, Oliveira F. The immune response to sand fly salivary proteins and its influence on leishmania immunity. Front Immunol. 2012;3:110. Epub 2012/05/18. doi: 10.3389/fimmu.2012.00110 ; PubMed Central PMCID: PMC3349933. 22593758 PMC3349933

[11] Kammoun-Rebai W, Bahi-Jaber N, Naouar I, Toumi A, Ben Salah A, Louzir H, et al. Human cellular and humoral immune responses to Phlebotomus papatasi salivary gland antigens in endemic areas differing in prevalence of Leishmania major infection. PLoS Negl Trop Dis. 2017;11(10):e0005905. Epub 2017/10/13. doi: 10.1371/journal.pntd.0005905 ; PubMed Central PMCID: PMC5638224. 29023574 PMC5638224

[12] Ben-Abid M, Galai Y, Habboul Z, Ben-Abdelaziz R, Ben-Sghaier I, Aoun K, et al. Diagnosis of Mediterranean visceral leishmaniasis by detection of Leishmania-related antigen in urine and oral fluid samples. Acta Trop. 2017;167:71–2. Epub 2016/12/27. doi: 10.1016/j.actatropica.2016.12.026 . 28017861

[13] Oliveira F, Kamhawi S, Seitz AE, Pham VM, Guigal PM, Fischer L, et al. From transcriptome to immunome: identification of DTH inducing proteins from a Phlebotomus ariasi salivary gland cDNA library. Vaccine. 2006;24(3):374–90. Epub 2005/09/13. doi: 10.1016/j.vaccine.2005.07.085 . 16154670

[14] Rohousova I, Volf P. Sand fly saliva: effects on host immune response and Leishmania transmission. Folia Parasitol (Praha). 2006;53(3):161–71. Epub 2006/11/24. . 17120496

[15] Rohousova I, Ozensoy S, Ozbel Y, Volf P. Detection of species-specific antibody response of humans and mice bitten by sand flies. Parasitology. 2005;130(Pt 5):493–9. Epub 2005/07/05. doi: 10.1017/s003118200400681x . 15991492

[16] Quinnell RJ, Soremekun S, Bates PA, Rogers ME, Garcez LM, Courtenay O. Antibody response to sand fly saliva is a marker of transmission intensity but not disease progression in dogs naturally infected with Leishmania infantum. Parasit Vectors. 2018;11(1):7. Epub 2018/01/06. doi: 10.1186/s13071-017-2587-5 ; PubMed Central PMCID: PMC5755305. 29301571 PMC5755305

[17] Vinhas V, Andrade BB, Paes F, Bomura A, Clarencio J, Miranda JC, et al. Human anti-saliva immune response following experimental exposure to the visceral leishmaniasis vector, Lutzomyia longipalpis. Eur J Immunol. 2007;37(11):3111–21. Epub 2007/10/16. doi: 10.1002/eji.200737431 . 17935072

[18] Barral A, Honda E, Caldas A, Costa J, Vinhas V, Rowton ED, et al. Human immune response to sand fly salivary gland antigens: a useful epidemiological marker? Am J Trop Med Hyg. 2000;62(6):740–5. Epub 2001/04/17. doi: 10.4269/ajtmh.2000.62.740 . 11304066

[19] Marzouki S, Ben Ahmed M, Boussoffara T, Abdeladhim M, Ben Aleya-Bouafif N, Namane A, et al. Characterization of the antibody response to the saliva of Phlebotomus papatasi in people living in endemic areas of cutaneous leishmaniasis. Am J Trop Med Hyg. 2011;84(5):653–61. Epub 2011/05/05. doi: 10.4269/ajtmh.2011.10-0598 ; PubMed Central PMCID: PMC3083729. 21540371 PMC3083729

[20] Teixeira C, Gomes R, Collin N, Reynoso D, Jochim R, Oliveira F, et al. Discovery of markers of exposure specific to bites of Lutzomyia longipalpis, the vector of Leishmania infantum chagasi in Latin America. PLoS Negl Trop Dis. 2010;4(3):e638. Epub 2010/03/31. doi: 10.1371/journal.pntd.0000638 ; PubMed Central PMCID: PMC2843637. 20351786 PMC2843637

[21] de Moura TR, Oliveira F, Novais FO, Miranda JC, Clarencio J, Follador I, et al. Enhanced Leishmania braziliensis infection following pre-exposure to sandfly saliva. PLoS Negl Trop Dis. 2007;1(2):e84. Epub 2007/12/07. doi: 10.1371/journal.pntd.0000084 ; PubMed Central PMCID: PMC2100374. 18060088 PMC2100374

[22] Abdeladhim M, Ben Ahmed M, Marzouki S, Belhadj Hmida N, Boussoffara T, Belhaj Hamida N, et al. Human cellular immune response to the saliva of Phlebotomus papatasi is mediated by IL-10-producing CD8+ T cells and Th1-polarized CD4+ lymphocytes. PLoS Negl Trop Dis. 2011;5(10):e1345. Epub 2011/10/13. doi: 10.1371/journal.pntd.0001345 ; PubMed Central PMCID: PMC3186761. 21991402 PMC3186761

[23] Oliveira F, Traore B, Gomes R, Faye O, Gilmore DC, Keita S, et al. Delayed-type hypersensitivity to sand fly saliva in humans from a leishmaniasis-endemic area of Mali is Th1-mediated and persists to midlife. J Invest Dermatol. 2013;133(2):452–9. Epub 2012/09/21. doi: 10.1038/jid.2012.315 ; PubMed Central PMCID: PMC3529997. 22992802 PMC3529997

[24] Vallur AC, Duthie MS, Reinhart C, Tutterrow Y, Hamano S, Bhaskar KR, et al. Biomarkers for intracellular pathogens: establishing tools as vaccine and therapeutic endpoints for visceral leishmaniasis. Clin Microbiol Infect. 2014;20(6):O374–83. Epub 2013/11/19. doi: 10.1111/1469-0691.12421 ; PubMed Central PMCID: PMC3977011. 24237596 PMC3977011

[25] Lane RP. The sandflies of Egypt (Diptera: Phlebotominae). Bull Brit Mus Nat Hist. 1986;52:1–35.

[26] Abdeladhim M, Kamhawi S, Valenzuela JG. What’s behind a sand fly bite? The profound effect of sand fly saliva on host hemostasis, inflammation and immunity. Infect Genet Evol. 2014;28:691–703. Epub 2014/08/15. doi: 10.1016/j.meegid.2014.07.028 ; PubMed Central PMCID: PMC4562216. 25117872 PMC4562216

[27] Lestinova T, Rohousova I, Sima M, de Oliveira CI, Volf P. Insights into the sand fly saliva: Blood-feeding and immune interactions between sand flies, hosts, and Leishmania. PLoS Negl Trop Dis. 2017;11(7):e0005600. Epub 2017/07/14. doi: 10.1371/journal.pntd.0005600 ; PubMed Central PMCID: PMC5509103. 28704370 PMC5509103

[28] Carvalho AM, Cristal JR, Muniz AC, Carvalho LP, Gomes R, Miranda JC, et al. Interleukin 10-Dominant Immune Response and Increased Risk of Cutaneous Leishmaniasis After Natural Exposure to Lutzomyia intermedia Sand Flies. J Infect Dis. 2015;212(1):157–65. Epub 2015/01/18. doi: 10.1093/infdis/jiv020 ; PubMed Central PMCID: PMC4539914. 25596303 PMC4539914

[29] Rohousova I, Volf P, Lipoldova M. Modulation of murine cellular immune response and cytokine production by salivary gland lysate of three sand fly species. Parasite Immunol. 2005;27(12):469–73. Epub 2005/11/01. doi: 10.1111/j.1365-3024.2005.00787.x . 16255746

[30] Lestinova T, Vlkova M, Votypka J, Volf P, Rohousova I. Phlebotomus papatasi exposure cross-protects mice against Leishmania major co-inoculated with Phlebotomus duboscqi salivary gland homogenate. Acta Trop. 2015;144:9–18. Epub 2015/01/20. doi: 10.1016/j.actatropica.2015.01.005 . 25597641

[31] Oliveira F, Giorgobiani E, Guimaraes-Costa AB, Abdeladhim M, Oristian J, Tskhvaradze L, et al. Immunity to vector saliva is compromised by short sand fly seasons in endemic regions with temperate climates. Sci Rep. 2020;10(1):7990. Epub 2020/05/16. doi: 10.1038/s41598-020-64820-9 . 32409684 PMC7224377

[32] Ribeiro JM, Katz O, Pannell LK, Waitumbi J, Warburg A. Salivary glands of the sand fly Phlebotomus papatasi contain pharmacologically active amounts of adenosine and 5’-AMP. J Exp Biol. 1999;202(Pt 11):1551–9. Epub 1999/05/07. . 10229701 10.1242/jeb.202.11.1551

[33] Katz O, Waitumbi JN, Zer R, Warburg A. Adenosine, AMP, and protein phosphatase activity in sandfly saliva. Am J Trop Med Hyg. 2000;62(1):145–50. Epub 2000/04/13. doi: 10.4269/ajtmh.2000.62.145 . 10761741

[34] Hasko G, Szabo C, Nemeth ZH, Kvetan V, Pastores SM, Vizi ES. Adenosine receptor agonists differentially regulate IL-10, TNF-alpha, and nitric oxide production in RAW 264.7 macrophages and in endotoxemic mice. J Immunol. 1996;157(10):4634–40. Epub 1996/11/15. . 8906843

[35] Nylen S, Sacks D. Interleukin-10 and the pathogenesis of human visceral leishmaniasis. Trends Immunol. 2007;28(9):378–84. Epub 2007/08/11. doi: 10.1016/j.it.2007.07.004 . 17689290

[36] Klein B, Zhang XG, Lu ZY, Bataille R. Interleukin-6 in human multiple myeloma. Blood. 1995;85(4):863–72. Epub 1995/02/15. . 7849308

[37] Rogers KA, Titus RG. Immunomodulatory effects of Maxadilan and Phlebotomus papatasi sand fly salivary gland lysates on human primary in vitro immune responses. Parasite Immunol. 2003;25(3):127–34. Epub 2003/08/13. doi: 10.1046/j.1365-3024.2003.00623.x . 12911520

[38] Costa DJ, Favali C, Clarencio J, Afonso L, Conceicao V, Miranda JC, et al. Lutzomyia longipalpis salivary gland homogenate impairs cytokine production and costimulatory molecule expression on human monocytes and dendritic cells. Infect Immun. 2004;72(3):1298–305. Epub 2004/02/24. doi: 10.1128/iai.72.3.1298-1305.2004 ; PubMed Central PMCID: PMC356034. 14977931 PMC356034

[39] Norsworthy NB, Sun J, Elnaiem D, Lanzaro G, Soong L. Sand fly saliva enhances Leishmania amazonensis infection by modulating interleukin-10 production. Infect Immun. 2004;72(3):1240–7. Epub 2004/02/24. doi: 10.1128/iai.72.3.1240-1247.2004 ; PubMed Central PMCID: PMC356033. 14977924 PMC356033

