# Supplementary material for: The human immune response to saliva of Phlebotomus alexandri, the vector of visceral leishmaniasis in Iraq, and its relationship to sand fly exposure and infection
Source: PLoS Negl Trop Dis. 2021 Jun 3;15(6):e0009378. doi: 10.1371/journal.pntd.0009378 (PMC8174707; doi:10.1371/journal.pntd.0009378)
Supplement: S1 Table — SGH: Salivary Gland Homogenates; AVL: asymptomatic visceral leishmaniasis (DOCX) [file pntd.0009378.s001.docx]

**S1** Table. Ratio IFN-γ to IL-10, IL-13 and IL-17

|  | **IFNg/IL-10** | **IFNg/IL-13** | **IFNg/IL-17** |
| --- | --- | --- | --- |
| **AVL-SGHAb+** | **0.1** | **0.13** | **0.55** |
| **AVL+SGHAb-** | **0.19** | **0.07** | **0.43** |
| **AVL+SGHAb+** | **0.35** | **0.2** | **6.12** |
| **AVL-SGHAb-** | **0.03** | **0.4** | **0.62** |

SGH: Salivary Gland Homogenates; AVL: asymptomatic visceral leishmaniasis
